# Dietary Application of Tannins as a Potential Mitigation Strategy for Current Challenges in Poultry Production: A Review

**DOI:** 10.3390/ani10122389

**Published:** 2020-12-14

**Authors:** Janghan Choi, Woo Kyun Kim

**Affiliations:** Department of Poultry Science, University of Georgia, Athens, GA 30602, USA; choij@uga.edu

**Keywords:** tannins, chickens, gut health, feed additives, antimicrobials, antioxidants

## Abstract

**Simple Summary:**

There are diverse challenges in the poultry production industry that decrease the productivity and efficiency of poultry production, impair animal welfare, and pose issues to public health. Furthermore, the use of antibiotic growth promoters (AGP) in feed, which have been used to improve the growth performance and gut health of chickens, has been restricted in many countries. Tannins, polyphenolic compounds that precipitate proteins, are considered as alternatives for AGP in feed and provide solutions to mitigate challenges in poultry production due to their antimicrobial, antioxidant, anti-inflammatory and gut health promoting effects. However, because high dosages of tannins have antinutritional effects when fed to poultry, determining appropriate dosages of supplemental tannins is critical for their potential implementation as a solution for the challenges faced in poultry production.

**Abstract:**

The poultry industry has an important role in producing sources of protein for the world, and the size of global poultry production continues to increase annually. However, the poultry industry is confronting diverse challenges including bacterial infection (salmonellosis), coccidiosis, oxidative stress, including that caused by heat stress, welfare issues such as food pad dermatitis (FPD) and nitrogen and greenhouse gasses emissions that cumulatively cause food safety issues, reduce the efficacy of poultry production, impair animal welfare, and induce environmental issues. Furthermore, restrictions on the use of AGP have exacerbated several of these negative effects. Tannins, polyphenolic compounds that possess a protein precipitation capacity, have been considered as antinutritional factors in the past because high dosages of tannins can decrease feed intake and negatively affect nutrient digestibility and absorption. However, tannins have been shown to have antimicrobial, antioxidant and anti-inflammatory properties, and as such, have gained interest as promising bioactive compounds to help alleviate the challenges of AGP removal in the poultry industry. In addition, the beneficial effects of tannins can be enhanced by several strategies including heat processing, combining tannins with other bioactive compounds, and encapsulation. As a result, supplementation of tannins alone or in conjunction with the above strategies could be an effective approach to decrease the need of AGP and otherwise improve poultry production efficiency.

## 1. Introduction

Poultry products including meat and eggs account for a significant part of global food production and constitute a protein staple throughout the world [1]. The United States Department of Agriculture (USDA) reported in 2016 that global egg production was approximately 70 million tons, and poultry meat production reached more than 100 million tons, which accounted for more than one-third of global meat production [2]. Moreover, global poultry production continues to increase annually [1]. However, there are many challenges in the poultry industry including: bacterial infection (salmonellosis); parasitic infection (coccidiosis); oxidative stress, including that caused by heat stress; welfare issues such as food pad dermatitis (FPD); and nitrogen and greenhouse gas emissions which can cause severe economic losses, threaten food safety and public health, impair animal welfare, and induce environmental pollution [2,3,4,5].

Antibiotic growth promoters (AGP) have been supplemented to chicken diets to improve growth performance and gut health, predominantly due to their antimicrobial effects and immunomodulatory functions in chickens [6,7]. However, because of the increased public concern about the transmission of antibiotic-resistant bacteria from poultry products, the use of AGP in poultry production has been banned or restricted in many countries [8,9,10]. In addition, some producers in the U.S. poultry industry have opted to entirely remove the use of antibiotics and instead raise chickens using “no antibiotics ever (NAE)” or “raised without antibiotics (RWA)” approaches [11,12]. As a consequence, the efficiency of poultry production has decreased due to increases in various bacterial and parasitic infections and reductions in the growth rate of chickens [13]. In addition, because there is no “magic bullet” that can replace AGP, some poultry producers are still using antibiotics in the U.S. and in many other countries, and the use of antibiotics for livestock animals in the world is expected to increase, possibly owing to population growth which is associated with a greater demand for livestock products in middle-income countries [14]. Therefore, it is essential to find alternatives to AGP, which must be cost-effective, eco-friendly and have antimicrobial and growth-promoting effects, without causing side effects (e.g., generation of resistant bacteria) to the animals and humans [15].

Tannins, defined as polyphenolic compounds that can precipitate proteins, are secondary metabolites, which are found in plants, seeds, bark, wood leaves and fruit skins and serve as plant defense mechanisms against predation [16]. High concentrations of tannins have been shown to have antinutritional effects in monogastric animals because tannins can decrease feed intake, nutrient digestibility and growth performance of chickens [17,18]. However, recently in poultry production, tannins have garnered a great deal of attention as an alternative for AGP because of their antimicrobial, antioxidants and anti-inflammation properties [19,20,21]. In addition, many tannins are considered sustainable feed additives, as they derive from byproducts of plant-based agriculture and industry. For example, chestnut tannins, which are already sometimes supplemented to poultry, are obtained by the distillation of wood that is used in the building industry [22]. However, the effects of tannins on the growth performance and gut ecosystem of the chickens are still inconsistent and their mode of action is unclear. Therefore, it is important to understand the chemical properties and biological effects of tannins to maximize the use of supplemental tannins in chickens. This review is mainly focused on the classification and bioavailability of tannins, the effects of distinct tannins on mitigating the challenges facing poultry production, and strategies to enhance the effects of tannins. 

## 2. Classification and Bioavailability of Tannins

**Figure 1:**
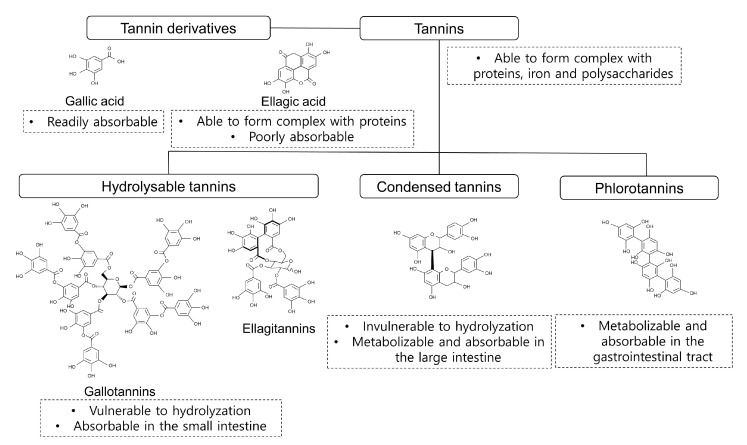
Classification and examples of molecular structure of tannins. Hydrolysable tannins and condensed tannins belong to plant tannins, and phlorotannins are found in brown algae.

Tannins, defined as polyphenolic compounds that have a protein precipitation capacity, exist in several different types with various molecular weights [23]. Plant tannins are classified into hydrolysable tannins (HT) with tannin derivatives (e.g., gallic acid and ellagic acid) and condensed tannins (CT) [24] (Figure 1). Additionally, phlorotannins (PT) are a third class of tannin unique to brown algae. Tannins have different bioavailabilities (absorbability), and their level of bioavailability varies depending on several factors, including the derivatives of each tannin (e.g., gallic acid and ellagic acid), their affinity to protein, molecular structure and molecular weight. The bioavailability of tannins is an important trait for their functionality and should be considered for tackling different issues in poultry production. Tannins with a low bioavailability potentially have better antimicrobial effects in chickens, whereas highly bioavailable tannins would be more beneficial as antioxidant and anti-inflammatory agents.

The HT including predominately gallotannins and ellagitannins (molecular weight 500 to 3000 Da) contain a polyol (normally glucose) as a central core, which is esterified with phenolic groups (e.g., gallic acid and ellagic acid) [25]. Under certain conditions (e.g., thermal processing, esterification, and acid or base treatment), HT can be hydrolyzed to yield free gallic acid and ellagic acid. Gallotannins produce one molecule of sugar and 9 to 10 molecules of gallic acid when hydrolyzed, whereas ellagitannins yield one molecule of sugar and several molecules of gallic acid and ellagic acid [26]. The unhydrolyzed HT are partially absorbable in the small intestine [27]. Gallic acid is readily absorbable into the blood stream [28], while ellagic acid has low bioavailability due to strong affinity for proteins and poor absorption [29]. Hence, HT include gallotannins and ellagitannins and have different bioavailability depends on the components and structure. 

The CT are defined as oligomeric or polymeric flavonoids containing flavan-3-olunits such as catechin, epicatechin, gallocatechin and epigallocatechin [24]. The CT exist in fruits (e.g., berries, pears and apples), forage legumes (e.g., lentils, black-eyed peas, chickpeas and red kidney beans), nuts, red and green grapes (and their juice and wine) [30,31,32]. The CT have higher molecular weight (1000—20,000 Da) and more complex structure compared to HT [24]. Unlike HT, CT are not vulnerable to hydrolyzation, which may imply low bioavailability in the gastrointestinal tract (GIT) of the chickens. This is because high molecular weight of tannins are hardly absorbable in the intestine [33]. Kahle et al. [34] reported that around 90% procyanidins (CT) were recovered in the distal ileum, which imply that most of CT can reach to the large intestine. The CT in the large intestine can be metabolized and absorbed by epithelial cells [35]. The CT have low bioavailability compared to HT and can be delivered to the large intestine of the chicken. 

The PT, algae-derived polyphenols, have molecular sizes ranging 126 Da to 650 kDa, but the majority of them are 10 and 100 kDa [36,37]. By using in-vitro models, Corona et al. [38] demonstrated that PT can be metabolized and absorbed in the upper GIT potentially due to low molecular weight of PT, and limited amounts of biologically active PT were delivered to the colon. In addition, the author also demonstrated that higher molecular weight of PT are less vulnerable to metabolization and absorption in the upper GIT [38]. Nwosu et al. [39] showed that PT can be metabolized and absorbed by the colon cells (e.g., Caco-2). Therefore, PT can be easily metabolized and absorbed in the GIT tracts due to their low molecular weights. However, more future in-vivo experiments are required to study bioavailability of PT in chickens. 

Taken together, tannins are classified into HT, CT and PT, and their bioavailability are depending on their components, structure and molecular weight. It is important to understand specific chemical properties each tannin prior to application in poultry diets. 

## 3. Traditional Viewpoints on Tannins as Antinutritional Factors in Poultry Production

While tannins were known as beneficial bioactive compounds in ruminants, they have been considered as antinutritional factors in poultry diets [16]. In the (GIT) of ruminants, tannins bind proteins under rumen pH (pH 5.5–7.0), inhibiting the microbial degradation of dietary proteins. Upon reaching the abomasum (pH 2.5–3.5), the non-covalent linkages between proteins and tannins are broken, and free protein can be absorbed by the host in the distal small intestine (pH 7.5) [40]. As such, tannins are known to increase ruminant’s protein utilization and decrease gas emission [41,42]. However, in chickens, tannins were considered as phytotoxins predominately due to their protein binding properties, which impair dietary protein digestion and decrease activity of digestive enzymes [43]. Moreover, tannins bind proline-rich, hydrophobic salivary proteins of chickens, forming complexes that are responsible for an astringent taste of the feed, and in turn lead to low palatability and decreased feed intake of chickens [44]. In addition, a high concentration of tannic acid (25 g/kg) showed toxicity to chickens by showing liver proteolytic activity in broiler chickens [45]. Lee et al. [46] reported that tannic acid impaired growth performance, hematological indices and plasma iron status in weaning pigs potentially because tannic acid can form complex with iron. However, these toxic properties of tannins were shown when tannins were included in the diets more than 7.5 g/kg (based on tannic acid, considered as the standard of HT), and many current studies proved that appropriate amounts of tannins (based on tannic acid; standard of tannins) ranging from to 0.5 g/kg to 5 g/kg in poultry could improve grow rate and gut health due to their potential antimicrobial, antioxidants and anti-inflammatory functions (Table 1). Therefore, while tannins were considered as anti-nutritional factors in the past, tannins at appropriate dosages have potentials to improve growth performance and gut health of chickens.

## 4. Challenges in Poultry Production and Potential Solution by Using Tannins

### 4.1. Effects of Tannins on Bacterial Infection (Salmonellosis)

There are many pathogenic bacteria in chickens and poultry products (meat and eggs), including *Salmonella Typhimurium*, *Escherichia coli* O157:H7, *Campylobacter jejuni*, *Clostridium perfringens*, enterohemorrhagic *Escherichia. coli* (EHEC), *Listeria monocytogenes*, *Arcobacter butzleri*, *Mycobacterium avium* subsp. *Paratuberculosis* and *Aeromonas hydrophila* [53]. These pathogenic bacteria not only impair gut health and growth rate of chickens, but are also a public health threat as foodborne diseases in humans [54]. Salmonellosis, one of the main food-borne diseases from poultry products, is induced by *S. Typhimurium* and *S. Enteritis* and causes, illness, morbidity and mortality in humans [55]. 

Diverse in-vitro studies showed that tannins and their derivatives showed bacteriostatic (inhibiting bacterial growth) and bactericidal (killing bacteria) effects on *Salmonella* spp. and other pathogens, as shown in Table 2. Potential mechanisms of antibacterial effects of tannins include (1) direct interactions with components in the cell wall to alter morphology of the cell wall and to increase membrane permeability of bacteria [24]; (2) decreasing activities of microbial enzymes [56]; and (3) depriving essential nutrients such as proteins and minerals (e.g., iron) for pathogenic bacteria [57,58,59]. In addition to bacteriostatic and bactericidal effects of tannins, many in-vitro studies reported that sub-lethal dosages of tannins also restricted pathogenicity of *Salmonella* spp. and other pathogens by inhibiting motility [60], quorum sensing [61] and biofilm formation [62] of pathogenic bacteria.

**Figure 2:**
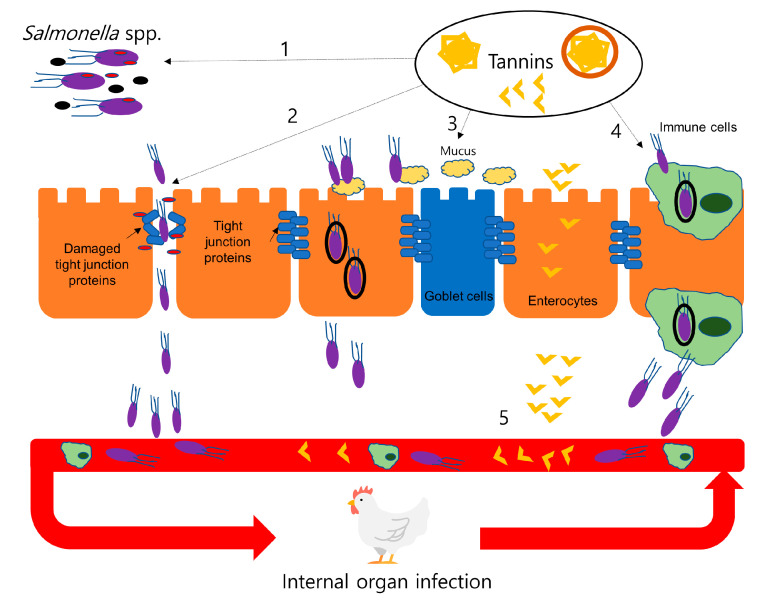
Systemic infection routes of *Salmonella* spp. and potential mechanisms of antibacterial actions of tannins in an in-vivo chicken model. Chickens can orally ingest *Salmonella* spp. from feed or their environment, and subsequently escape the small or large intestine [75]. (1) Tannins and tannin derivatives (tannins, hydrolyzed tannins, and tannin-protein complexes) are known to inhibit the growth of *Salmonella* spp. in the intestine and decrease quorum sensing of the bacteria [61]. Lipopolysaccharides (LPS) of *Salmonella* spp. can impair intestinal barrier function, which allows *Salmonella* spp. to pass the paracellular pathways of the intestine [76,77]. (2) Tannins and tannin derivatives may improve gut barrier integrity by neutralizing LPS or decreasing expression of cytokines, which can impair tight junction proteins [78]. (3) and (4) *Salmonella* spp. also utilized mucus and immune cells to invade epithelial cells [71]. Tannins and their derivates potentially modulate expression and morphology of immune cells and mucus. *Salmonella* spp. can invade epithelial cells in diverse pathways and enter blood circulation and finally colonize in internal organs (e.g., liver, kidney, spleen, etc.) and muscles in chickens [79]. (5) Tannins also can enter the blood circulation and potentially show antimicrobial effects and modulate immune cells to attenuate internal organ infection by *Salmonella* spp. in chickens [80,81].

In addition to their antibacterial effects, tannins can control systemic infection of *Salmonella* spp. by beneficially modulated components of gut ecosystem. *Salmonella* spp. can enter blood circulation via paracellular and transcellular pathways and use immune cells to enter enterocytes to be distributed in the internal organs and muscles in chickens (Figure 2). Many studies reported that tannins altered expression and functionality of tight junction proteins [68], mucus [69,70], and immune cells [71,72] of chickens. Nevertheless, supplemental tannins have not typically translated to antimicrobial effects against *Salmonella* spp.in in-vivo studies. For example, while Van Parys et al. [73] reported that whereas HT extract of sweet chestnut woods showed strong antimicrobial effects (minimum inhibitory concentration: 25–50 μg/mL and minimum inhibitory concentration: 100 μg/mL) against *S. Typhimurium* isolated from pigs under *in-vitro* conditions, the inclusion of 3000 mg/kg HT in the pig feed did not reduce the *Salmonella* spp. concentration in feces, intestine and internal organs of pigs inoculated 10^7^ colony forming units (CFU)/mL of *S. Typhimurium* strain at four days post-inoculum. Similarly, Kubena et al. [51] reported that tannic acid (7.5 or 15 g/kg in the feed) did not modulate the salmonella concentration in cecal content of broiler chickens inoculated with 10^4^ CFU of *S. typhimurium.* Potential explanations for such findings include (1) tannins were degraded by host or microbial enzymes or absorbed before they reached to the lower intestine where most of the *Salmonella* spp. and other pathogenic bacteria propagate; (2) tannins formed complexes with components of feedstuffs (polysaccharides and proteins) or endogenous proteins, which inhibits antimicrobial effects of tannins; and (3) experimental factors including the low dosages of tannins, the high dosages of salmonella inoculum or short period of the experiments were possibly obstacles to diminish antimicrobial effects of tannins in in-vivo models [73]. In contrast, Jamroz et al. [74] reported that 1000 mg/kg of sweet chestnut tannins reduced the number of *E. coli* and coliforms bacteria in the small intestine on 28 d; however, growth performance of the tannin-fed birds was decreased in this study.

While many in-vitro studies showed that tannins showed antimicrobial effects against *Salmonella* spp. and other pathogens, the optimal antimicrobial dosages in chickens have not yet been fully determined. Therefore, dose-specific antimicrobial effects of tannins in chickens, as well as their mode of actions, warrant further investigation.

### 4.2. Effects of Tannins on Coccidiosis

Coccidiosis, which is a parasitic disease induced by protozoa of the family *Eimeridae*, is one of the most prevalent and detrimental enteric diseases in poultry production [82]. The nine identified *Eimeria* species in chickens includes *E. acervulina*, *E. brunetti*, *E. maxima*, *E. necatrix*, *E. praecox*, *E. mitis*, *E. tenella*, *E. mivati* and *E. hagani* to date [83,84]. *Eimeria* spp. infect and multiply within the mucosal epithelial layers in the different parts of the GIT through the fecal-oral route [85]. Coccidiosis can result in reduced growth rate and gut barrier integrity and induce inflammation, diarrhea, hemorrhaging, and even mortality in broiler chickens, negatively influencing the efficacy of poultry production and welfare [86,87]. The negative effects of coccidiosis on gut health of chickens are closely related to oxidative stress, as *Eimeria* infections cause lipid peroxidation and excessive production of reactive oxygen species (ROS) in chickens [88]. Furthermore, coccidiosis is closely associated with an enteric infectious disease, necrotic enteritis, which is predominately induced by *C. perfringens* with the presence of *Eimeria* spp. [89,90]. To cope with coccidiosis for the poultry industry, prophylactic coccidiostats and anticoccidial drugs have been supplemented in poultry diets [91]. However, resistance for all currently available drugs have been documented, making it imperative to discover novel drug alternatives that induce limited resistance and effectively control coccidiosis in broiler chickens [92]. To find alternatives for prophylactic coccidiostats and anticoccidial drugs, bioactive compounds including prebiotics [93], plant extracts [94], organic acids [95], essential oils [96], lipids (fatty acids) [97], minerals (e.g., zinc) [98] and nitro compounds [99] have been studied in chickens. 

Tannins are known to have anticoccidial effects because tannins can form complexes with parasitic enzymes and metal ions, which are essential for *Eimeria* spp. and can stimulate immune system against of the chickens [100,101,102]. Tonda et al. [47] reported that the dietary supplementation of 500 mg/kg of gallnut tannic acid extract reduced total oocyst number in excreta, and 500 mg/kg of tannic acid or gallnut tannic acid extract decreased intestinal lesion scores in broilers infected with *Eimeria* spp. Furthermore, the authors showed that gallnut tannic acid extract enhanced feed conversion ratio of cocci-vaccinated birds, which possibly implies that gallnut tannic acid extract improved protective immunity following coccidiosis vaccination [47]. Supplemental chestnut HT and quebracho CT tannins reduced *Eimeria* spp. oocyst shedding and parasitic bacterial diarrhea and attenuated negative effects of coccidiosis via immunomodulating and anti-inflammatory effects in rabbits [103]. In broiler chickens, Kaleem et al. [104] showed that administration of *Emblica officinalis* derived tannins improved growth performance and showed immunostimulatory properties and enhanced protective immune system. The beneficial effects of tannins on gut health of chickens infected with *Eimeria* spp. are closely associated with antioxidant properties of tannins, which can restore an *Eimeria*-damaged gastrointestinal [105]. A study by [106] demonstrated that supplementation of grape seed proanthocyanidin extract, rich in CT, enhanced growth performance and attenuated clinical symptoms, potentially by improving antioxidant capacity in chickens infected with *Eimeria tenella*. In contrast, Mansoori and Modirsanei [107] showed that supplemental tannic acid (10 g/kg) numerically increased D-xylose absorption in chickens vaccinated against coccidiosis followed by challenging with *Eimeria* spp., however, supplemental tannic acid increased the total number of oocysts in excreta, indicating that high dosages of tannins can attenuate the efficiency of anticoccidial vaccines and impair appropriate development of immune system against coccidiosis in chickens. The discrepancy may be attributed to different sources and concentrations of tannins and dissimilar experimental conditions (e.g., challenge dosages of *Eimeria* spp.).

Although many studies reported the potential benefits of supplemental tannins in broiler chickens infected *Eimeria* spp., more comprehensive studies are required (1) to study the mechanisms under anticoccidial effects of tannins in chickens; (2) to elucidate mechanisms of the beneficial effects of tannins on the gut health of chickens infected with *Eimeria* spp.; (3) to investigate the effects of tannins in a necrotic enteritis challenge model by using inoculum of *Eimeria* spp. and *C. perfringens*; and (4) to find appropriate concentrations and types of tannins against coccidiosis in chickens. 

### 4.3. Effects of Tannins on Oxidative Stress, Including that Caused by Heat Stress

**Figure 3:**
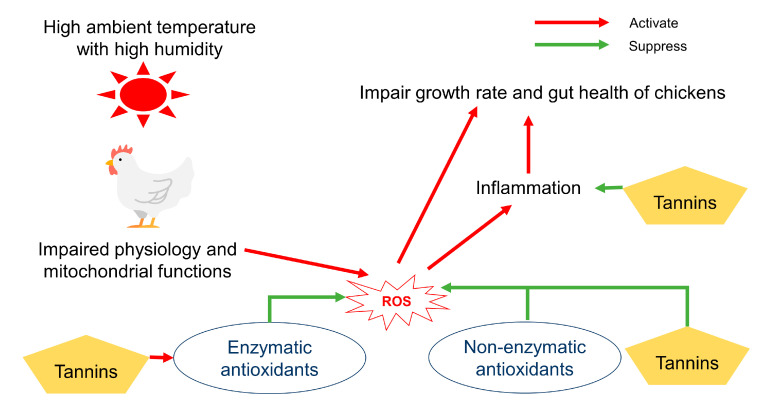
Potential mechanisms for the alleviating effects of tannins in heat-stressed chickens. High ambient temperature with high humidity increase core body temperature of chickens, and this can result in impaired physiology and mitochondrial functions, which lead to excessive reactive oxygen species (ROS) [127]. Tannins, polyphenolic compounds, directly scavenge ROS like non-enzymatic antioxidants [128], and also, tannins can increase activities of antioxidant enzymes such as superoxide dismutase (SOD), glutathione peroxidase (GPX), and glutathione reductase (GR) in broiler chickens. The ROS cause inflammation that can impair growth rate and gut health of chickens and tannins also have anti-inflammatory function [124].

Heat stress is one of the major obstacles in the poultry industry because heat stress negatively impacts growth performance, gut health, meat quality, and welfare of chickens [108,109]. A potential reason for negative effects of heat stress on chickens is closely associated with excessive production of ROS via accelerated metabolic reactions due to mitochondrial respiration [110,111,112]. Under normal conditions, enzymatic and non-enzymatic antioxidants can neutralize ROS and maintain the balance between oxidants and antioxidants [113,114] (Figure 3). However, if there is an imbalance between oxidants and antioxidants in chickens, excessively produced ROS can impair gut health and induce inflammation, which results in decreased growth performance of chickens [115]. Antioxidants including plant extracts [116,117], L-carnitine [118], vitamin C [119,120], vitamin E [121], and selenium [122] have been studied to alleviate heat stress and oxidative stress. 

Tannins are believed to relieve or attenuate effects of oxidative stress, including that caused by heat stress by scavenging ROS and modulating enzymatic antioxidants in animals (Figure 3) [116]. The reducing power of tannins including proanthocyanidins, catechins, epicatechin, and procyanidin from grade seeds is approximately 20 times higher than vitamin E and 50 times higher than vitamin C [123]. A study from Sahin et al. [124] reported that 200 or 400 mg/kg of epigallocatechin-3-gallate (the ester of epigallocatechin and gallic acid) from green tea relieved oxidative stress by controlling the hepatic nuclear transcription factors such as nuclear factor κ-light-chain-enhancer of activated B cells (NF-κB) and nuclear factor (erythroid-derived 2)-like 2 (Nrf2) in heat stressed quails. Furthermore, 10 mg/kg supplementation of tannic acid improved fatty acid profile (decreased monosaturated fatty acids) in breast muscle of chickens under heat stress [52]. Ramnath and Rekha [125] showed that supplementation of *Brahma Rasayana* containing various sorts of tannins enhanced activities of enzymatic antioxidants including superoxide dismutase (SOD), glutathione peroxidase (GPx), glutathione reductase (GR), and reduced glutathione (GSH) in blood of chickens raised at a cold temperature. Moreover, the inclusion of grape (*Vitis vinifera*) pomace, rich in CT, enhanced antioxidant enzyme activities (GPx and SOD) and intestinal morphology, and increased relative weight of bursa of Fabricius and thymus in the heat-stressed broiler chickens [126]. In a diquat-induced mouse model (oxidative stress model), tannic acid improved intestinal morphology, activated the antioxidative pathway by reducing protein expression of Kelch like-ECH-associated protein 1 (KEAP1) and enhancing protein expression of nuclear factor erythroid 2-related factor 2 (NRF2), as well as modulated intestinal barrier function in the jejunum. Thus, supplementing appropriate concentrations of tannins potentially would be an effective strategy to attenuate oxidative stress in heat-stressed birds. 

### 4.4. Effects of Tannins on Food Pad Dermatitis (FPD)

Foot pad dermatitis (FPD) is defined as a condition that induces lesions on the plantar surface of the footpads in growing chickens [129]. The FPD causes severe economic losses in poultry production because paws are the third most crucial economic part in broiler chickens, and FPD can impair growth rate, gut health, and welfare of chickens [129,130]. Thøfner et al. [131] showed that FPD and systemic bacterial infections are closely correlated because pathogens can invade to the chickens through damaged epithelium on the foot pads [132]. Litter moisture and litter quality are the most two crucial features that cause FPD among varied factors such as bedding materials and depth, drinkers and nutrient deficiencies [129,133,134]. In addition, increased excreta viscosity has been known to increase the occurrence and severity of FPD in chickens by modulating litter moisture and litter quality [135]. 

Tannins can relieve the incidence and severity of FPD by enhancing fecal consistency (e.g., fecal dry matter contents) and litter quality [16]. Cengiz et al. [48] reported that 2000 mg/kg supplementation of tannic acid reduced the incidence and severity of FPD in broiler chickens without affecting growth performance, litter quality and intestinal viscosity of chickens. Moreover, 700 mg/kg and 2000 mg/kg of tannin-rich sweet chestnut wood extract increased fecal dry matter contents in chickens [136]. In addition, antimicrobial, antioxidant and anti-inflammatory properties of tannins probably helped to attenuate the incidence and severity of FPD in broiler chickens because oxidative stress and inflammation can exacerbate the severity and consequences of FPD in chickens [137,138]. Thus, supplementation of tannins has potential to reduce severity and incidence of FPD in broiler chickens by enhancing fecal consistency and litter quality. 

### 4.5. Effects of Tannins on Nitrogen Excretion and Emissions of Noxious and Greenhouse Gases

Reactive nitrogen species (ammonia, nitrous oxide, and other oxides of nitrogen) and sulfur-containing compounds (hydrogen sulfide and sulfur dioxide) are produced in poultry production facilities and cause environment pollution and greenhouse gases such as carbon monoxide, carbon dioxide and methane [139,140,141,142]. Factors affecting the production of nitrogen compounds and detrimental gases include types of feedstuffs, manure conditions, and housing accessories (bedding and heating materials) [143,144]. Nitrogen and greenhouse gases are produced in the livestock animals due to enteric fermentation and manure fermentation, and ruminants contribute most of the livestock greenhouse gas production because of high fermentation rate in the rumen [140]. Although poultry production is not a major contributor of noxious or greenhouse gases, the continued growth of poultry production coupled with higher intensity of excretion and emission per unit compared to other species, more attention is needed to find strategies to mitigate the production of nitrogen excretion and emissions of noxious and greenhouse gases [145].

It is well-established that tannins improve N utilization and decrease methane production in ruminant animals [25,146]. The potential mechanisms underlying effects of tannins on mitigation of emissions of nitrogen and greenhouse gases are inhibiting growth of methanogens, reducing protozoal-associated methane production, and decreasing fiber fermentation [25]. Although chickens (monogastric animals) have distinct GIT from ruminants, tannins may alter gut health and microbiota and improve N utilization in chickens, thereby potentially reducing nitrogen and methane emissions. Ahmed and Yang [147] reported that supplementation of by-products of *Punica granatum* (fruit), which contains HT such as ellagitannins, punicalagin, punicalin and pedunculagin, decreases the emission of ammonia and methanethiol from excreta of broiler chickens. Moreover, a study by Bostami et al. [139] demonstrated that supplementation of fermented pomegranate byproducts, containing ellagitannins, reduced gas emission (ammonia and hydrogen sulfide) from excreta in broiler chickens, potentially via reducing microbial activity and pH of excreta. However, 2000 mg/kg of tannic acid from chestnut wood did not affect ammonia volatilization in broiler chickens. Hence, more studies are required to identify suitable sorts and dosages of tannins to reduce nitrogen excretion and emissions of noxious and greenhouse gases during poultry production. 

### 4.6. Effects of Tannins on Growth Performance, Immune System, Gut Microbiota, Gut Ecosystem and in Chickens Raised under General Conditions 

While some authors showed that low concentrations (0.5 g/kg to 5 g/kg) of tannic acid improved growth performance (shown in Table 1), the others reported that supplementation of different sources of tannins at low dosages (0.5 to 5 g/kg) did not affect growth performance [48,136] and even showed negative effects on growth performance of the birds [74]. These differences may be a result of different tannin sources, supplementation period, or specific experiment conditions (e.g., genetics of chickens, temperature and abundance of pathogens in the living conditions). 

However, supplemental tannins at appropriate dosages can improve immune system, gut ecosystem and gut microbiota of chickens raised under general conditions. Ramah et al. [148] reported that whereas the high dosage of tannic acid (30 g/kg diet) have negative impacts on immune system by decreasing relative (cluster of differentiation) CD4+, CD8+, CD4+CD8+ and γδ+ cell populations in thymus, spleen, and cecal tonsils and by reducing cytokine mRNA expression in spleen cells, the low dosage of tannic acid (0.5 g/kg) enhanced CD4+CD8+ subpopulations and γδ+ cells in spleen and CD4+CD8+ subpopulations and B cells in cecal tonsils and increasing mRNA expression of IFN-γ in broiler chickens. A study by Karaffová et al. [149], reported that tannins were beneficial to maintain components of mucosal immunity of chickens via upregulating immunoglobulins A and mucin 2. Erlejman et al. [150] also demonstrated that CT can combine with receptors of tumor necrosis factor-α (pro-inflammatory cytokines) to inhibit inflammation, which implies that tannins directly modulate immune system without eliciting antimicrobial and antioxidant properties.

Diaz Carrasco et al. [151] reported that a blend of chestnut (HT) and quebracho tannins (CT) modulated diversity cecal microbiota of chickens and decreased genus *Bacteroides* and increased certain members of order Clostridiales predominately in the families Ruminococcaceae and Lachnospiraceae. A study by Koo and Nyachoti [152] demonstrated that tannic acid positively modulated microbial metabolites in pigs fed oxidized oil. Selective antimicrobial effects of tannins would beneficially modulate microbiome of animals [153]. Viveros et al. [154] also suggested that tannins probably can have prebiotic effects via stimulating the proliferation of the beneficial bacteria.

Microbiome modulating effects of tannins may partially explain the gut health promoting effects of the chickens because microbiota of the chicken is closely associated with the gut ecosystem of chickens [155]. Moreover, an in-vitro study by Brus et al. [19] reported that chestnut tannins stimulated proliferation of enterocytes and enhanced antioxidative properties of the chicken small intestinal epithelial cells. Bilić-Šobot et al. [156] demonstrated that HT decreased production of cell debris in the large intestine of pigs, which leaded to decreased production of skatole, which belongs to the indole family. Together, supplemental tannins have the potential to improve growth performance, gut microbiota and gut ecosystem in broiler chickens, even in the absence of challenge models.

## 5. Strategies to Maximize the Effects of Supplemental Tannins in the Chickens

### 5.1. Heat Process on Tannins

Some in-vitro studies showed that heat processed HT had better antimicrobial and antioxidant properties than unprocessed HT [143,144]. This would be because heat processing could partially hydrolyze tannic acid and release gallic acid molecules, and these newly produced gallic acid and galloyl groups had enhanced antimicrobial and antioxidant effects compared to the fresh tannic acid [157]. González et al. [145] also reported that thermal process of *hamamelis virginiana* containing gallotannins and CT improved efficacy of antioxidant properties for inhibiting lipid oxidation. However, because CT are hardly hydrolyzed, enhanced antioxidant of heat-processed *hamamelis virginiana* probably due to hydrolyzation of gallotannins in *hamamelis virginiana* rather than hydrolyzation of CT. Thus, while in-vitro studies found that heat process of tannins could improve their functional properties (e.g., antioxidant and antimicrobial effects) compared to unprocessed HT, it is unknown yet whether heat-processed tannins have more beneficial effects on animal models.

### 5.2. Co-Supplementation of Tannins with other Bioactive Compounds

Supplementation of tannins with other bioactive compounds could be more beneficial to chickens than supplementing tannins alone for several reasons: (1) complexed form with proteins or polysaccharides of tannins inhibit tannins to form a complex with endogenous and dietary proteins and metal ions; (2) distinct properties of bioactive compounds can show synergistic effects to antimicrobial effects against both gram negative and positive bacteria; (3) different bioactive compounds affect gut health in different ways, which can lead to synergistic effects in animals; and (4) by providing more than two bioactive compounds, pathogenic bacteria are hard to generate resistant system against diverse bioactive compounds. Table 3 shows that tannins have potentials to show synergistic effects with other bioactive compounds.

Probiotics are living microorganisms which beneficially affect the host animals by enhancing animal’s intestinal microbial balance [158]. Probiotics may have different mode of actions from tannins to inhibit the growth of pathogenic bacteria and to improve gut health of chickens. Probiotics can improve gut integrity by modulating immune system and maintaining microflora of chickens and tannins, while tannins can show antioxidant and anti-inflammatory properties [159]. However, one of the concerns of using probiotics with tannins could be that tannins may show antimicrobial effects against probiotics. However, Khalil [160] showed that gallic acid and catechin polyphenols did not inhibit the growth of *Streptococcus thermophilus* (probiotics), and Pacheco-Ordaz et al. [153] reported that catechin, gallic, vanillic, ferulic and protocatechuic acids selectively inhibit the growth of pathogenic bacteria without decreasing viability of probiotics. More studies are required to establish synergistic effects and mechanisms of tannins and probiotics in *in-vivo* chicken models.

Organic acids, known as strong antimicrobials, are organic compounds with acidic properties. Tannins inhibit the growth of pathogens predominately by inhibiting activities of microbial enzymes and modulating bacterial membrane, but organic acids penetrate bacterial cell wall, and bacteria have to spend a lot of energy to pump out hydrogen molecules, which causes bacterial death [162,163]. Furthermore, organic acids are known to improve intestinal morphology and gut barrier integrity by being energy sources for epithelial cells, which may imply that organic acids with tannins can show synergistic effects [164,165,166]. Thus, combination of tannins and organic acids can have synergistically increased antimicrobial effects and gut health promoting effects due to different mode of actions.

### 5.3. Supplementation of Combined or Encapsulated Form of Tannins

If tannins are combined with proteins, polysaccharides and ions before being included in the chickens feed, the tannins in complexes would not bind dietary and endogenous proteins and metal ions in chickens. The tannin complexes would be loose in the high pH (>7.0) in the intestine of chickens, and proteins in the tannin complexes can be degraded by digestive enzymes in the small intestine of chickens [40,167]. However, Lee et al. [46] showed that supplementation of albumin-tannin complexes still decreased growth performance and negatively modulated microbiota, hematological indices and plasma iron status of weaning piglets. The delivery of tannin-protein or polysaccharide complexes in the GIT of chickens, and effects of diverse dosages of supplemental tannin complexes on growth rate and gut health of chickens should be further investigated.

Encapsulation techniques, which offer a physical barrier for bioactive compounds and separate the core material from the environment until their release, have obtained a lot of attention in the livestock industry because encapsulation can maximize the efficacy of feed additives that have stability, cost and environmental issues. Encapsulation has been applied to various vulnerable feed additives such as essential oils [96,168,169], probiotics [170], organic acids [171], bacteriophages [172], zinc [173] and exogenous enzymes [174]. Diverse materials including proteins [96,175], lipids [171], carbohydrates (starch) [169], and polysaccharides [172,176] have been used to encapsulate bioactive compounds.

Encapsulation techniques can be applied to decrease side effects and maximize benefits of tannins in chickens. Encapsulation of tannins can depress the protein binding capacity of tannins, which decreases feed intake by making astringent taste and digestibility of proteins and induces dietary and endogenous protein losses [177]. In addition, more tannins can be delivered to small or large intestine where many pathogens propagate by decreasing bioavailability for absorption in the upper GIT of chickens. Adejoro et al. [178] showed that lipid-encapsulated acacia tannin extracts reduced methane production and enhanced neutral detergent fiber digestibility in sheep. A study by Wang et al. [179] reported that microencapsulated tannic acid improved intestinal morphology in duodenum, increased expression of ileal nutrient transporters (sodium-dependent neutral amino acid transporter; B^0^AT1 and peptide transporter 1; PepT1) and modulate microbiota without affecting growth performance of weaned piglets even though ileal maltase activity and gene expression of jejunal sodium-dependent glucose transporter 1 (SGLT1) was reduced. Future studies are required to develop effective encapsulated tannins and to determine appropriate dosages of encapsulated tannins for chickens.

## 6. Conclusions

There are various kinds of tannins, which have different bioavailability in chickens. Depends on the sorts and dosages of tannins, tannins can elicit detrimental effects on growth performance and gut ecosystem of the broiler chickens or can beneficially modulate growth performance and gut ecosystem of the broiler chickens. In addition, heat processing, co-supplementation with other bioactive compounds and encapsulation potentially enhance the beneficial biological effects of tannins. In conclusion, supplementation of tannins alone or in conjunction with those strategies have large potential to alleviate challenges, replace AGP and improve production efficiency in poultry productions.

## Figures and Tables

**Table 1:** Effects of different dosages of tannic acid on the broiler chickens.

Dosages of Tannic Acid	Outcomes	References
0.5 g/kg	Improved growth performance and immune system in broiler chickens challenged with *Eimeria* spp.	[47]
2 g/kg	Improved foot pad dermatitis of the chickens without affecting growth performance	[48]
5 g/kg	Improved growth performance and decreased lipid oxidation.	[49]
5 g/kg	Increased growth performance, modulated cecal microbial metabolites and decreased cecal pH.	[50]
7.5 and 15 g/kg	Decreased growth performance and did not inhibit the growth of *Salmonella typhimurium* in broiler chickens challenged with *S. typhimurium*.	[51]
10 g/kg	Attenuated fatty acid profile of breast but decreased growth performance in the heat stressed chickens.	[52]
25 g/kg	Decreased growth performance and showed liver toxicity by inducing liver proteolytic activity	[45]

**Table 2:** In-vitro antimicrobial effects tannins against diverse pathogenic bacteria.

Tannins Sources	Strains	Results and Conclusions	References
Ellagitannins from Chestnut wood Gallotannins from Tara and Sumach (Gall nuts) Condensed tannins from Quebaracho and *Calliandra calothyrsus* Flavanol gallates from Tea and *Acacia nilotica*	*Salmonella Typhimurium*	All of the tannins inhibited the growth of *S. Typhimurium.*	[63]
Tannic acid Gallic acid	*S. Typhimurium*	Tannic acid and gallic acid had bactericidal effects and gallic acid had higher bactericidal effects than tannic acid	[64]
Condensed tannins extracted from tree leaves viz. babool (*Acacia nilotica*), jamun (*Eugenia jambolana*), peepal (*Ficus religiosa*), subabul (*Leucaenia leucocephala*) and guajava (*Psidium guajava*)	*Escherichia coli* *Staphylococcus aureus* *S. enteritis Enterococcus faecalis*	All of the selected five condensed tannins inhibited the growth of the four pathogenic bacteria	[65]
Tannin extracts from *Cytinus hypocistis* and *C. ruber*	*S. aureus* *S. epidermidis* *E. faecium* *Pseudomonas aeruginosa* *Klebsiella pneumoniae*	Tannin extracts from *C. hypocistis* and *C. ruber* showed antibacterial and antibiofilm activities against gram positive and negative human pathogens.	[66]
Chestnut tannins (80% hydrolysable tannins) Quebracho tannins (75% condensed tannins)	*Clostridium perfringens*	Both hydrolysable and condensed showed antimicrobial effects against *C. perfringens* and neutralized its cytotoxicity.	[67]

**Table 3:** Effects of tannins with other bioactive compounds on the chickens.

Tannins	Other Bioactive Compounds	Outcomes	References
100 mg/kg tannic acid extract	Probiotics (1 × 10^4^ spores/kg *Bacillus coagulans*)	Improved feed conversion ratio of coccidiosis vaccinated broilers.	[47]
240 mg/kg tannic acid	Organic acids (420 mg/kg lactic, 480 mg/kg butyric acid and 480 mg/kg acetic acid)	Decreased *S. enteritis* horizontal transmission in broiler chickens	[161]
Chestnut tannins	Saturated short medium chain fatty acids (C4:0 to C12:0)	Showed strong antimicrobial effects in in-vitro conditions and did not affect growth performance and meat quality of in-vivo chicken models.	[22]
